# Pandemic Influenza A/H1N1pdm in Italy: Age, Risk and Population Susceptibility

**DOI:** 10.1371/journal.pone.0074785

**Published:** 2013-10-07

**Authors:** Stefano Merler, Marco Ajelli, Barbara Camilloni, Simona Puzelli, Antonino Bella, Maria Cristina Rota, Alberto Eugenio Tozzi, Maurizio Muraca, Marcello Meledandri, Anna Maria Iorio, Isabella Donatelli, Caterina Rizzo

**Affiliations:** 1 Predictive Models for Biomedicine & Environment, Bruno Kessler Foundation, Trento, Italy; 2 Department of Infectious, Parasitic and Immune-mediated Diseases, National Institute of Health, Rome, Italy; 3 Department of Medical and Surgical Specialties and Public Health, University of Perugia, Perugia, Italy; 4 National Centre for Epidemiology, Surveillance and Health Promotion, National Institute of Health, Rome, Italy; 5 Bambino Gesù Children Hospital and Research Institute, Rome, Italy; 6 San Filippo Neri Hospital, Rome, Italy; University of Hong Kong, Hong Kong

## Abstract

**Background:**

A common pattern emerging from several studies evaluating the impact of the 2009 A/H1N1 pandemic influenza (A/H1N1pdm) conducted in countries worldwide is the low attack rate observed in elderly compared to that observed in children and young adults. The biological or social mechanisms responsible for the observed age-specific risk of infection are still to be deeply investigated.

**Methods:**

The level of immunity against the A/H1N1pdm in pre and post pandemic sera was determined using left over sera taken for diagnostic purposes or routine ascertainment obtained from clinical laboratories. The antibody titres were measured by the haemagglutination inhibition (HI) assay. To investigate whether certain age groups had higher risk of infection the presence of protective antibody (≥1∶40), was calculated using exact binomial 95% CI on both pre- and post- pandemic serological data in the age groups considered. To estimate age-specific susceptibility to infection we used an age-structured SEIR model.

**Results:**

By comparing pre- and post-pandemic serological data in Italy we found age- specific attack rates similar to those observed in other countries. Cumulative attack rate at the end of the first A/H1N1pdm season in Italy was estimated to be 16.3% (95% CI 9.4%-23.1%). Modeling results allow ruling out the hypothesis that only age-specific characteristics of the contact network and levels of pre-pandemic immunity are responsible for the observed age-specific risk of infection. This means that age-specific susceptibility to infection, suspected to play an important role in the pandemic, was not only determined by pre-pandemic levels of H1N1pdm antibody measured by HI.

**Conclusions:**

Our results claim for new studies to better identify the biological mechanisms, which might have determined the observed pattern of susceptibility with age. Moreover, our results highlight the need to obtain early estimates of differential susceptibility with age in any future pandemics to obtain more reliable real time estimates of critical epidemiological parameters.

## Background

After the detection of the new A/H1N1 pandemic influenza virus (A/H1N1pdm) in late April 2009 [1], in Mexico and United States, which indicated the beginning of the 2009 pandemic, the World Health Organization (WHO) declared the pandemic over in August 2010 [2].

In Italy only one major epidemic wave was observed, with most cases recorded from September to December 2009. Overall, from August 2009 to April 2010, approximately 5.6 million (9.3% of the Italian population) of medically attended influenza-like illness (ILI) cases were reported to the sentinel surveillance system Influnet (including a total of 2,000 laboratory 2009 A/H1N1pdm confirmed cases from May to October 2009), including 1,106 confirmed cases admitted to hospital for serious conditions and 260 deaths [3]. Epidemiological surveillance showed that during the first season of the pandemic the A/H1N1pdm infected many more school age children than adults [3].

Several serological studies, conducted in different countries worldwide, have estimated overall attack rates and age-specific attack rates [4], comparing pre- and post-pandemic samples [5]. In Europe serial seroprevalence studies were carried out [6],[7], [8]–[19]. Similar serial seroprevalence studies were conducted in the United States [20], Canada [21], [22], New Zealand [23], Australia [24] China [25] and Hong Kong [26], [27]. A common pattern in all the above described studies was the relatively low overall attack rate and the surprisingly low attack rate observed in elderly compared to that observed in children and young adults [28]. However, the biological and social factors determining the observed pattern of risk of infection were and still are to be deeply understood. Among possible factors we hypothesized: *i*) age-specific characteristics of the contact network might have determined differential age-specific risk of infection, e.g. much lower in elderly with respect to children and young adults [4], [26]; *ii*) pre-pandemic immunity might have conferred a certain level of herd immunity in the population, thus limiting virus transmission, especially in elderly [6], [29].

The aim of this paper is twofold: first, to assess whether estimates of overall attack rate and age-specific risk of infection in Italy comply with those obtained by other countries worldwide; second, to assess whether factors *i*) and *ii*) above described, which surely have had an impact, allow explaining the observed pattern of spread in Italy, in terms of age-specific attack rates and incidence over time.

Early in the pandemic, age-specific susceptibility to infection was suspected to play an important role [30]–[32]. As differential susceptibility to infection accounts for effects induced by pre-pandemic immunity, answering the previous questions will clarify whether age-specific susceptibility to infection is fully determined by pre-pandemic immunity.

## Methods

### Pre-pandemic sera

The level of immunity against A/H1N1pdm was determined pooling data data derived from a previous seroepidemiological study conducted on 587 leftover sera, collected in 2004 with a set of data derived from a set of 565 pre-pandemic sera collected in 1993 from the Reference Laboratory for Influenza of the Umbria region in central Italy. Sera collected in 2004 were specimens taken for diagnostic purposes or routine ascertainment obtained from clinical laboratories representative by age and gender of the Italian population. [33], These specimens were collected anonymously and only age, gender, geographic area and date of sampling were recorded for each sample. Sera from individuals known to be affected by an immunodepressive condition, by an acute infection, or to have recently undergone a blood transfusion were excluded. No other information about health status or symptoms was recorded at the time of blood sampling. These sera were tested for antibody to A/H1N1pdm by HI using standard methods as previously described [33].

Sera collected in 1993 were left over sera from one hospital laboratory in the Umbria region collected for seroepidemiological study purpose and stored at −20°C until tested.

Antibody titres against the pandemic A (H1N1) 2009 virus (A/California/7/2009 strain) of these sera were measured by the same HI assay used for the sera collected in 2004 [33], using a standard microtitre method [34] (see laboratory techniques section).

Pooled data were reanalyzed in the present study to estimate pre-pandemic age specific seroprotective levels and age specific risk of infection. We considered 6 age groups (0–4, 5–14, 15–24, 25–44, 45–64, ≥65 yrs) similar to those in which influenza like illness surveillance data are reported in Italy within the National sentinel surveillance system for Influenza (Influnet) [35]. All ages considered in this study refers to the age of individuals in 2009.

### Post-pandemic sera

Left over serum samples, taken for diagnostic purposes or routine ascertainment, were obtained from 7 diagnostic laboratories located in 3 different Italian regions, between August and September 2010 (i.e. before the start of the 2010/2011 influenza season in Italy). These specimens were collected anonymously and only age, gender, geographic area and date of sampling were recorded for each sample. Sera from individuals known to be affected by an immunodepressive condition, by an acute infection, or to have recently undergone a blood transfusion were excluded. No other information about health status or symptoms was recorded at the time of blood sampling. Serum samples were stored at the National Center for Influenza (NIC) of the Istituto Superiore di Sanità (ISS) at −20°C until use.

To estimate A/H1N1pdm antibody prevalence in the serum samples, we determined a total sample size of 1,400 sera with approximately 200 samples in each of the following 6 age groups: 0–4, 5–14, 15–24, 25–44, 45–64, ≥65 yrs. We used the same method described in [6]. Thus, with a sample size of 200, the 95% CIs for the estimated prevalence within each age group would be 2.4–9.0 for a 5% prevalence and 42.9–57.1 for a 50% prevalence. We excluded from the analysis sera from individuals born after the pandemic. All ages considered in this study refers to the age of individuals in 2009.

### Ethical approval

All samples tested were left over sera obtained at the point of discard. Samples were anonymised before testing, removing any link to any epidemiological or patient identifiable data, and for this reason informed consent is not necessary according to the ethical requirements of the Italian Ministry of Health and to the local clinical governance at each centre.

### Laboratory Techniques

Antibody titres against the pandemic A (H1N1) 2009 virus (A/California/7/2009 strain) were measured by the haemagglutination inhibition (HI) assay, using a standard microtitre method [34]. All sera were treated with receptor-destroying enzyme (RDE – Sigma-Aldrich, Italy) to remove non-specific inhibitors of hemagglutination. Briefly, 4 volumes of RDE were added to 1 volume of each serum (e.g. 0.4 ml RDE +0.1 ml serum) and incubated overnight in a 37°C water-bath. The following day, 0.5 ml of 1.5% sodium citrate solution were added to each tube and incubated in a 56°C water-bath for 30 minutes, to inactivate any remaining RDE. This procedure therefore resulted in a tenfold dilution of each serum. Serial 2-fold dilutions of treated sera from 1∶10 were then mixed with 4 haemagglutinin units of the new pandemic A/H1N1pdm, using live egg-grown A/California/4/09 virus, and after incubation at room temperature for 1 h, with 0.5% turkey erythrocytes. HI antibody titers ≥40, were considered protective according to EMEA criteria [36], also for the new pandemic virus and not representing antibody cross-reactive with previous circulating A/H1N1 viruses. Moreover, HI antibody titers ≥10, were considered.

### Age specific risk of infection

To investigate whether certain age groups had higher risk of infection, the prevalence of protective antibody (HI titre ≥1∶40) was calculated in pre- and post- pandemic serological data in the age groups considered (0–4, 5–14, 15–24, 25–44, 45–64, ≥65 yrs) with the relative 95% CI. Differences between pre and post pandemic prevalence were evaluated using binomial test.

Risk of infection by age group was defined as the difference between pre- and post-pandemic values.

As the sample size does not allow us to estimate the risk of infection for 1-year age brackets, we computed risk of infection on rolling windows of 25 post-pandemic samples for each age group considered, after having corrected post-pandemic serological data to account for pre-pandemic immunity [26]. Specifically, post-pandemic positive samples in every age group were randomly converted in pre-pandemic positive ones with probability proportional to the observed age specific pre-pandemic seroprevalence. In order to estimate average values and 95% CI, this procedure was repeated 1000 times, where the ordering of participants within each one-year age bracket and the assignment of pre-pandemic immunity to individuals testing positive after the pandemic were randomly sampled.

The same analysis was repeated by assuming that HI titre of 1∶10 is enough to guarantee protection level.

### Age specific susceptibility to infection

To estimate age-specific susceptibility to infection we used an age-structured SEIR model. In the model, the population is divided into four classes: susceptible (individual that can acquire the infection), latent (individual that acquired the infection but that are not able to transmit the disease yet), infectious (individuals able to transmit the disease) and recovered (individuals that are immune to the disease). In addition, each classes is divided into four age groups respecting the age classes of ILI surveillance system, i.e. 0–4 years, 5–14 years, 15–64 years, 65+ years. The model is described by the following equation system:
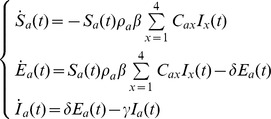
where *S_a_ (t), E_a_ (t)* and *I_a_ (t)* represent the number of susceptible, latent and infectious individuals of age group *a* at time *t* respectively. ρ_a_ is the susceptibility to infection of individuals in age group *a*. *β* is the transmission rate; *C_ax_* is the contact matrix representing the average number of contacts between individuals in age group *a* with individuals in the age group *x*, taken from the literature [37]–[39]; 1/*δ* is the average duration of the latent period; 1/*γ* is the average duration of the infectious period. According to the literature, we assume 1/*δ* = 1.5 days and 1/*γ* = 1.2 days, resulting in a serial interval of 2.7 days [30], [31], [40], [41]. As regards the contact matrix, we use three different matrices describing contact mixing patterns by age in Italy as available in the literature. Specifically we denote the three scenarios by: CM1, Italian matrix taken from [39]; CM2, Big-Italy matrix taken from [38]; CM3, Italian Polymod matrix taken from [37].

The parameters that we aim to estimate with model fit are five: the transmission rate *β*, the parameters characterizing age-specific susceptibility in the age groups 5–14 years, 15–64 years, 65+ years (assuming the susceptibility of age group 0–4 years equal to 1 to avoid over-parameterization), and the initial number of infected individuals I_0_ (which are distributed into classes *E_a_ (0), I_a_ (0)* according to age structure, length of latent and of infectious period). Parameter estimates are obtained by fitting the model to age specific 2009 A/H1N1 weekly incidence of A/H1N1pdm infections over time, from the reopening of schools after summer vacations (week 37, 2009) to the end of the epidemic (week 1, 2010). The A/H1N1pdm weekly incidence over time in the four age groups was estimated by assuming it to be proportional to ILI incidence over time as reported to the Influenza National Sentinel Surveillance system (Influnet) multiplied for the weekly fraction of cases positive testing for A/H1N1 and by rescaling the resulting incidence in order to obtain the same fraction of infected population in each age group at the end of the pandemic as resulting by the analysis of the collected sera.

For all three choices of contact matrices, the model fitting procedure was performed under four different assumptions: i) assuming that HI titre of 1∶40 guarantees immunity to influenza and initializing simulations assuming no fully protected individuals at the beginning of the pandemic ii) assuming that HI titre of 1∶10 guarantees immunity to influenza and initializing simulations assuming no fully protected individuals at the beginning of the pandemic, iii) HI titre of 1∶40 guarantees immunity to influenza and initializing simulations by assuming pre-existing full immunity in the different age groups as obtained by the analysis of pre-pandemic sera, iv) HI titre of 1∶10 guarantees immunity to influenza and initializing simulations by assuming pre-existing full immunity in the different age groups as obtained by the analysis of pre-pandemic sera. Unless otherwise stated, results refer to assumption i) (i.e., protection occurring at HI titre ≥40 and no fully immune individuals at the beginning of the pandemic).

## Results and Discussion

### Pre-pandemic serology

**Figure 1:**
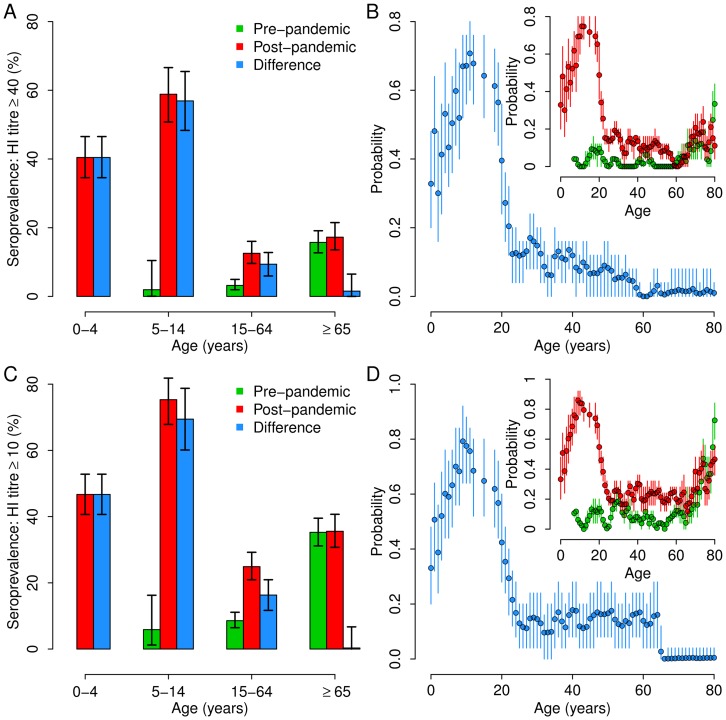
Serolgy and risk of infection by age. (A) Pre-pandemic seroprevalence (green), post-pandemic seroprevalence (red) and difference between post- and pre- seroprevalence (blue). Vertical bars represent 95% CI. An individual is considered to be seropositive when HI titre is ≥40 (B) Average (blue points) and 95% CI (vertical blue bars) of the probability distribution of the final fraction of infected individuals by age in moving windows of 25 study participants. The inset refers to pre- and post- pandemic data (colors as in panel A). An individual is considered to be seropositive when HI titre is ≥40 (C) As A, but considering seropositive individuals having HI titre ≥10. (D) as B, but considering seropositive individuals having HI titre ≥10.

Using HI assay 1,152 of the 1,172 available sera were tested. By reanalyzing pre-pandemic serological data, comprising 1,152 serum samples, we found very low levels of seroprotection (protection assumed to occur for HI titre ≥40) against the pandemic virus in all age groups with exception of the ≥65 age group (see Table 1 and Figure 1a). Specifically, we found that the pre-pandemic fraction of protected individuals was 2.0%, 7.2%, 1.5%, 2.7%, 16.4% in the age groups 5–14; 15–24; 25–44; 45–64; ≥65, respectively. No pre-pandemic serological data were available for the age group 0–4. Figure 1c shows the same analysis but assuming protection when HI titre ≥10.

**Table 1:** Seroprevalence of pre and post pandemic sera by age-group, using the haemoagglutination inhibition (HI) method and assuming individuals to be seropositive when HI titre is ≥40.

Age Group	Pre-pandemic samples (N = 1,152)		Post-pandemic samples (N = 1,236)		p-value
	number of positive/total number of samples	% HI ≥40 (95% CI)	number of positive/total number of samples	% HI ≥40 (95% CI)	
0–4	-	-	110/272	40.4 (34.5–46.5)	
5–14	1/50	2.0 (0, 10.6)	93/158	58.9 (50.8, 66.6)	<0.0001
15–24	9/124	7.2 (3.4, 13.3)	17/48	35.4 (22.2, 50.5)	<0.0001
25–44	3/203	1.5 (0.3, 4.2)	21/177	11.9 (7.5, 17.6)	<0.0001
45–64	7/258	2.7 (1.1, 5.5)	16/212	7.5 (4.4, 12.0)	0.0003
65+	85/517	16.4 (13.3, 19.9)	62/369	16.8 (13.1, 21.0)	0.48
Total	105/1,152	9.1 (7.5, 10.9)	319/1,236	25.8 (23.4,28.3)	

### Post-pandemic serology

Using HI assay 1,436 of the 1,439 sera available were analyzed. Of the 1436 left over serum samples collected, 1236 were stratified by specific age-groups, since 200 subjects were born after the pandemic. As expected, a very low fraction of subjects with protective antibody HI titres (HI≥40) was found in children born after the pandemic (1%, 95% CI 0.1–3.6). The fraction of protected individuals increases from pre-school ages is equal to 40.4% in 0–4 yrs group, and increases to 58.9% in 5–14 yrs group, and then decreases sharply in subjects belonging to the 15–24, 25–44, 45–64, age groups: 35.4%, 11.9%, and 7.5%, respectively. Finally, the level of seroprotection against the pandemic virus increases to 16.8%, in the ≥65 age group (see Table 1 and Figure 1a). Figure 1c shows the same analysis but assuming protection when HI titre ≥10.

### Age specific risk of infection

The difference between pre- and post-pandemic levels of seroprotection against the pandemic virus was significant (according to binomial test) in all age groups with the exception of the ≥65 age-group (see Table 1). Risk of infection was estimated to be 40.4% (95% CI: 34.6%–46.5%), 56.9% (95% CI: 48.3%–65.55%), 28.2% (95% CI: 13.9%–42.5%), 10.9% (95% CI: 5.8%–16%), 4.9% (95% CI: 0.8%–8.9%), and 1.5% (95% CI: 0%–6.5%), in the 0–4, 5–14, 15–24, 25–44, 45–64 and ≥65 age groups, respectively.

Figure1b shows the risk of infection as obtained by computing averages on moving windows of 25 analyzed sera. Results confirm that the percentage of subjects with protective antibody is lower in pre-school ages with respect to school ages. A sharp drop in risk of infection in ages older than school age (about 20 years) clearly arises, followed by a plateau for middle ages, before another drop in older adults (about 50 years). The same procedure was applied to pre- and post-pandemic data (see inset of Figure 1b).

Similar qualitative results, although with higher seropositive rates, were found by assuming protection when HI titre ≥10 (see Figure 1c, d).

### Cumulative infection attack rate

By using estimated values (difference between pre- and post-pandemic serology) in the 0–4, 5–14, 15–24, 25–44, 45–64 and ≥65 age groups (Figure 1a), cumulative attack rate in the Italian population was estimated to be 16.7%. By using smoothed values of the risk of infection by age (Figure 1b), cumulative attack rate was estimated to be 16.3% (95% CI 9.4%–23.1%). By assuming protection when HI titre ≥10 the cumulative attack rate was estimate to be 20.1% (95% CI: 12.3%–27.9%).

### Age specific susceptibility to infection

**Figure 2:**
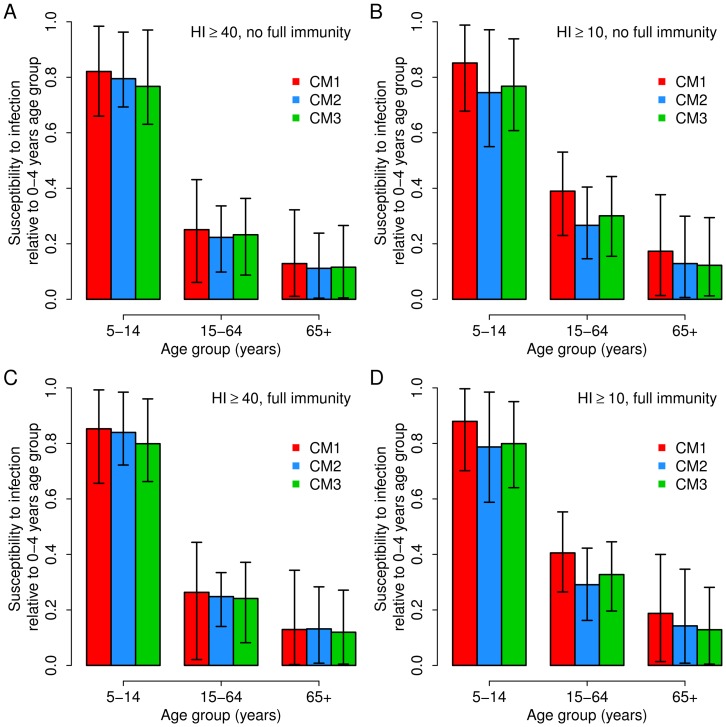
Relative susceptibility to infection. (A) Average value of the age-specific susceptibility to infection in age groups 5–14 years, 15–64 years and 65+ years, relative to that of the class 0–4 years. Vertical lines represent 95% CI. Colors refer to the contact matrix assumed, namely red for CM1 [39], blue for CM2 [38] and green for CM3 [37]. In this panel we assume that individuals are seropositive when HI titre is ≥40; in addition we assume that at the beginning of the simulations there are no fully immune individuals. (B) as A, but we assume that individuals are seropositive when HI titre is ≥10. (C) as A, but assuming pre-existing full immunity in the different age groups as obtained by the analysis of pre-pandemic sera. (D) as B, but assuming pre-existing full immunity in the different age groups as obtained by the analysis of pre-pandemic sera.

By assuming protection when HI titre ≥40 and no fully protected individuals at the beginning of the pandemic, average and 95% prediction intervals of model fit are in excellent agreement with those of the rescaled influenza incidence in the four age groups, irrespectively of the assumed contact matrix (see Figure S1). The resulting estimates of the basic reproduction number are R_0_ = 1.44 (95% CI: 1.38–1.5) when contact matrix CM1 is assumed, R_0_ = 1.45 (95% CI: 1.39–1.51) for CM2 and R_0_ = 1.45 (95% CI: 1.38–1.51) for CM3 (see also Table 2). Therefore, the estimate of R_0_ are very stable with respect to the choice of the contact matrix. In addition, the estimated values are in good agreement with the values found in the literature for the 2009 H1N1 pandemic in Italy (average R_0_ = 1.38) [31], [42]. Differently from what can be expected by simply looking at seroprevalence data by age, we found that the age group 0–4 was the most susceptible to infection; in fact, for each choice of the contact matrix, we found a decreasing trend of susceptibility to infection by age (see Figure 2a). This is due to the fact that mixing pattern by age are far from being homogeneous and, on the contrary, are highly assortative in children and adolescent [37]–[39]. Our results are in good agreement with those reported in [43], where the authors find the same monotone decrease in susceptibility to infection by age; moreover they estimate the susceptibility of 65+ years-old individuals is estimated to be around four times higher than 0–4 years age group as we found in this work.

**Table 2:** Estimation of the basic reproduction number R_0_ under different assumptions on immunity and mixing patterns.

Assumptions on immunity	Contact matrix	Average R_0_ (95% CI)
HI titre is ≥40, no fully protected individuals	CM1	1.44 (1.38–1.5)
	CM2	1.45 (1.39–1.51)
	CM3	1.45 (1.38–1.51)
HI titre is ≥10, no fully protected individuals	CM1	1.48 (1.42–1.53)
	CM2	1.48 (1.42–1.53)
	CM3	1.48 (1.43–1.54)
HI titre is ≥40, pre-existing full immunity	CM1	1.52 (1.42–1.63)
	CM2	1.53 (1.43–1.65)
	CM3	1.53 (1.43–1.65)
HI titre is ≥10, pre-existing full immunity	CM1	1.55 (1.46–1.66)
	CM2	1.55 (1.46–1.66)
	CM3	1.56 (1.47–1.66)

All four scenarios about immunity we analyzed gave rise to qualitatively similar results in terms of model fit (see Figure S2, S3 and S4), estimated reproduction number (see Table 2) and of age-specific susceptibility to infection (see Figure 2b, c and d); results are not sensitive with changes in the assumptions on the initial distribution of cases.

## Conclusions

After the first season of circulation of the A/H1N1pdm in the community, the highest increase in seropositivity rate to the new virus with respect to pre-pandemic values was found among children (5–14 yrs of age), resulting in an infection attack rate of 56.9%. On the contrary, the lowest significant increase in the seroprotection rate after the A/H1N1pdm was found among subjects aged 45–64 yrs (infection attack rate: 4.8%), whereas no substantial changes were observed among the elderly aged 65+ yrs (infection attack rate: 0.4%,). Qualitatively, our results are similar to those observed in several countries worldwide and previously reported in the literature. In European countries serial seroepidemiological studies were conducted in United Kingdom [6], [12], where a considerable increase in HI antibody titers in children living in metropolitan areas from 1.8% to 23% (0 to 4 years) and from 3.7% to 46% (5 to 14 years), was shown, and similar results were found in Scotland [44]. In Germany [8]–[10], [15], [16], in Greece Maltezou, 2011 2205/id}, France [17], Sweden [18] and Norway [11] results showed increasing antibody titers mostly in younger age groups, but also in the elderly [11]. With regard to overseas countries, similar serial seroprevalence studies were conducted in the US where an overall increase of seroprevalence from 6% to 21% was found with the highest prevalence observed among children aged 0 to 19 years, followed by over- 80-year-olds, while no increase in seroprevalence was observed among the 70- to 79-year-olds. Similar results were also obtained in New Zealand [24] and in Hong Kong where a study, using paired sera, showed a decreasing trend of infection rates by age [26].

All above described studies confirm the common pattern arising worldwide, that is the relatively low overall attack rate and the surprisingly low attack rate observed in elderly compared to that observed in children and young adults [4]–[6], [8]–[11], [20], [23], [24], [26], [28], [44]–[46].

We estimated that about 16% of the population was infected during the first season of virus circulation, a value similar to that obtained in other countries. Moreover, we estimated R_0_ to be 1.41 on average (95% CI 1.37–1.48); similar independent estimates were obtained for UK [31] and Italy [31], [42]. In this study we were unable to discriminate between vaccinated and unvaccinated individuals; however, pandemic vaccine coverage in Italy was about 1% in the general population [3] suggesting that the vaccination have probably not affected our results.

Modeling results allow ruling out the hypothesis that only age-specific characteristics of the network of contacts and levels of pre-pandemic immunity are responsible for the observed age-specific risk of infection. This means that age-specific susceptibility to infection, which early in the pandemic was suspected to play an important role [30]–[32], was not only determined by pre-pandemic levels of H1N1pdm antibody measured by HI. We estimated a high susceptibility to infection in individuals aged less than 18 years, followed by a drasticdecline in adult ages, to values lower than those estimated in [32]. These results claim for new immunological studies to test biological hypothesis in order to explain the observed pattern of susceptibility to infection with age. For instance, might vaccination against seasonal influenza have generated partial protection, especially in elderly? Is it possible that exposure to previously circulating influenza viruses A/H1N1 might have generated partial cross-protection, undetectable by measuring antibody titres against the pandemic A/H1N1pdm virus?

Given the crucial role played by age-specific susceptibility to infection in determining the observed pattern of spread, in terms of timing and impact, our results highlight the need to obtain early estimates of different age specific susceptibility to infection and more reliable real time estimates of critical epidemiological parameters. These results are crucial in order to define and better target Public Health intervention measures to be implemented during a pandemic situation.

## Supporting Information

Figure S1 **Model fit to rescaled weekly incidence data, assuming to be seropositive when titre is ≥40 and no pre-existing full immunity.** Average model prediction (colored line) and 95% CI (colored shaded area) and rescaled weekly incidence (black dots) with 95% CI (vertical black lines) in the four age groups. (A) Predictions obtained by assuming contact matrix CM1 [39]. (B) Predictions obtained by assuming contact matrix CM2 [38]. (C) Predictions obtained by assuming contact matrix CM3 [37].
(TIF)

Figure S2 **Model fit to rescaled weekly incidence data, assuming to be seropositive when titre is ≥10 and no pre-existing full immunity.** Average model prediction (colored line) and 95% CI (colored shaded area) and rescaled weekly incidence (black dots) with 95% CI (vertical black lines) in the four age groups. (A) Predictions obtained by assuming contact matrix CM1 [39]. (B) Predictions obtained by assuming contact matrix CM2 [38]. (C) Predictions obtained by assuming contact matrix CM3 [37].
(TIF)

Figure S3 **Model fit to rescaled weekly incidence data, assuming to be seropositive when titre is ≥40 and pre-existing full immunity as derived from the analysis of pre-pandemic sera.** Average model prediction (colored line) and 95% CI (colored shaded area) and rescaled weekly incidence (black dots) with 95% CI (vertical black lines) in the four age groups. (A) Predictions obtained by assuming contact matrix CM1 [39]. (B) Predictions obtained by assuming contact matrix CM2 [38]. (C) Predictions obtained by assuming contact matrix CM3 [37].
(TIF)

Figure S4 **Model fit to rescaled weekly incidence data, assuming to be seropositive when titre is ≥10 and pre-existing full immunity as derived from the analysis of pre-pandemic sera.** Average model prediction (colored line) and 95% CI (colored shaded area) and rescaled weekly incidence (black dots) with 95% CI (vertical black lines) in the four age groups. (A) Predictions obtained by assuming contact matrix CM1 [39]. (B) Predictions obtained by assuming contact matrix CM2 [38]. (C) Predictions obtained by assuming contact matrix CM3 [37].
(TIF)
