# Supplementary figures and images for: Pandemic Influenza A/H1N1pdm in Italy: Age, Risk and Population Susceptibility
Source: PLoS One. 2013 Oct 7;8(10):e74785. doi: 10.1371/journal.pone.0074785 (PMC3792117; doi:10.1371/journal.pone.0074785)

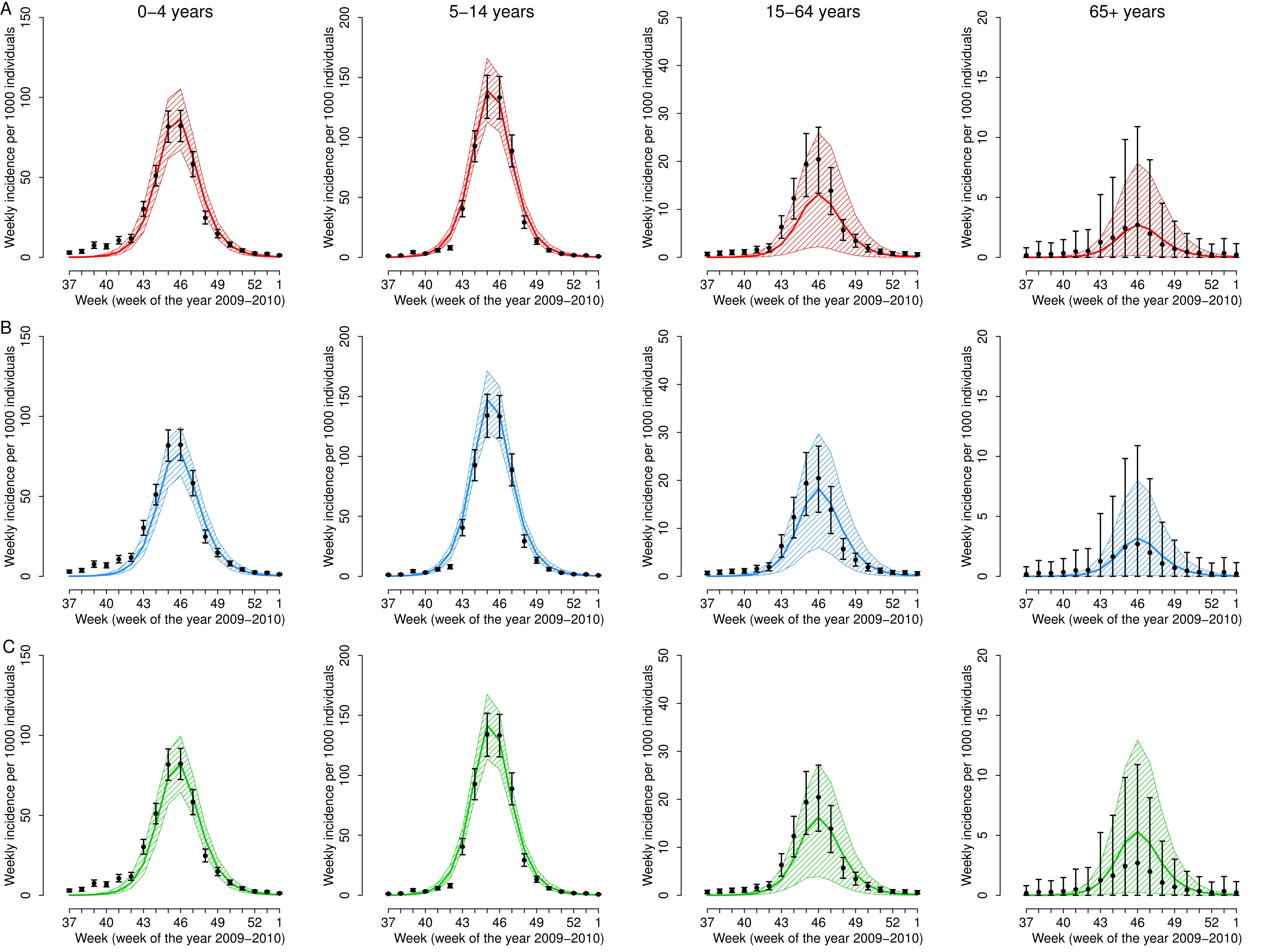

Supplement: Figure S1 — Model fit to rescaled weekly incidence data, assuming to be seropositive when titre is ≥40 and no pre-existing full immunity. Average model prediction (colored line) and 95% CI (colored shaded area) and rescaled weekly incidence (black dots) with 95% CI (vertical black lines) in the four age groups. (A) Predictions obtained by assuming contact matrix CM1 [39]. (B) Predictions obtained by assuming contact matrix CM2 [38]. (C) Predictions obtained by assuming contact matrix CM3 [37]. (TIF) [file pone.0074785.s001.tif]

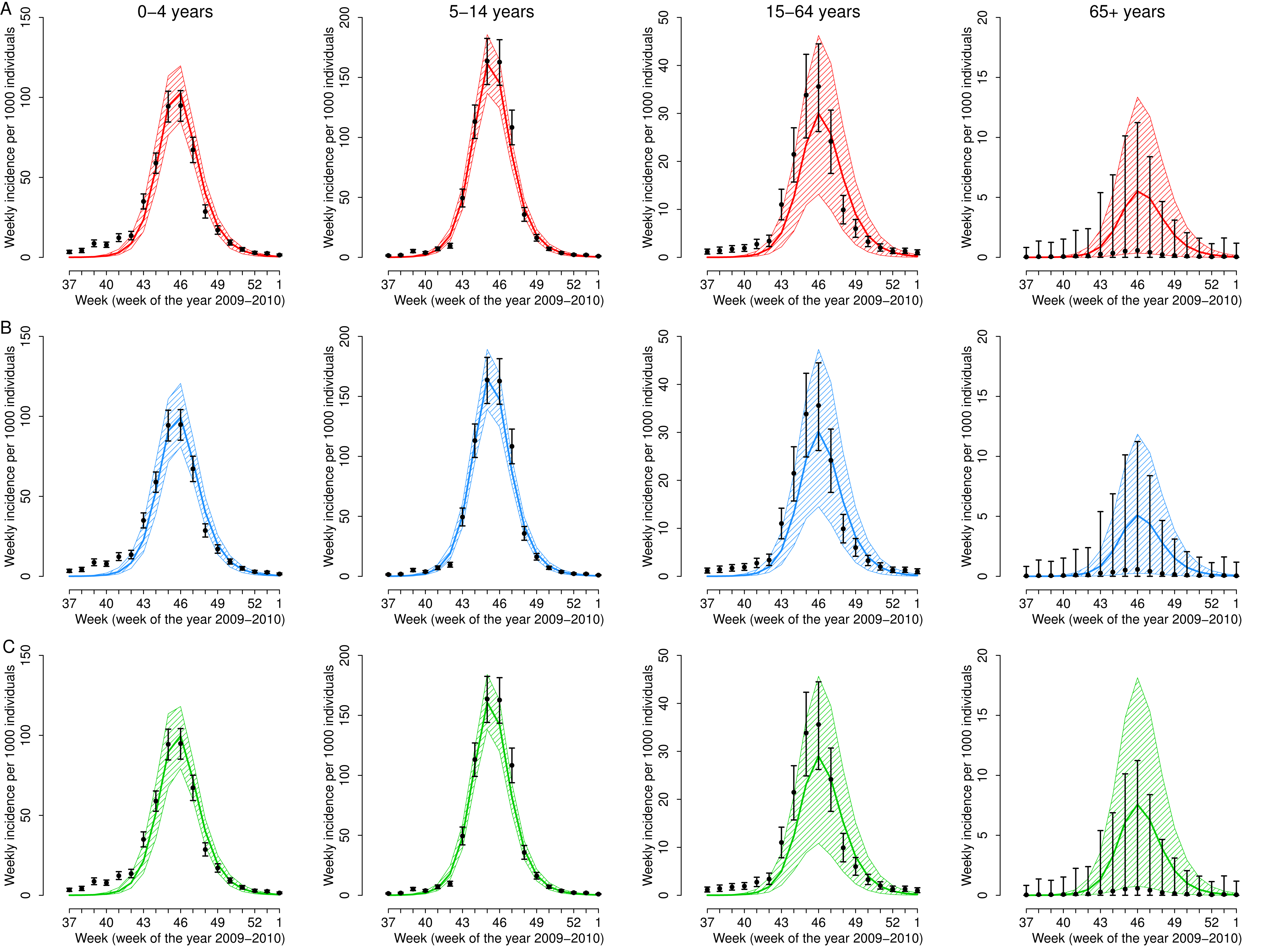

Supplement: Figure S2 — Model fit to rescaled weekly incidence data, assuming to be seropositive when titre is ≥10 and no pre-existing full immunity. Average model prediction (colored line) and 95% CI (colored shaded area) and rescaled weekly incidence (black dots) with 95% CI (vertical black lines) in the four age groups. (A) Predictions obtained by assuming contact matrix CM1 [39]. (B) Predictions obtained by assuming contact matrix CM2 [38]. (C) Predictions obtained by assuming contact matrix CM3 [37]. (TIF) [file pone.0074785.s002.tif]

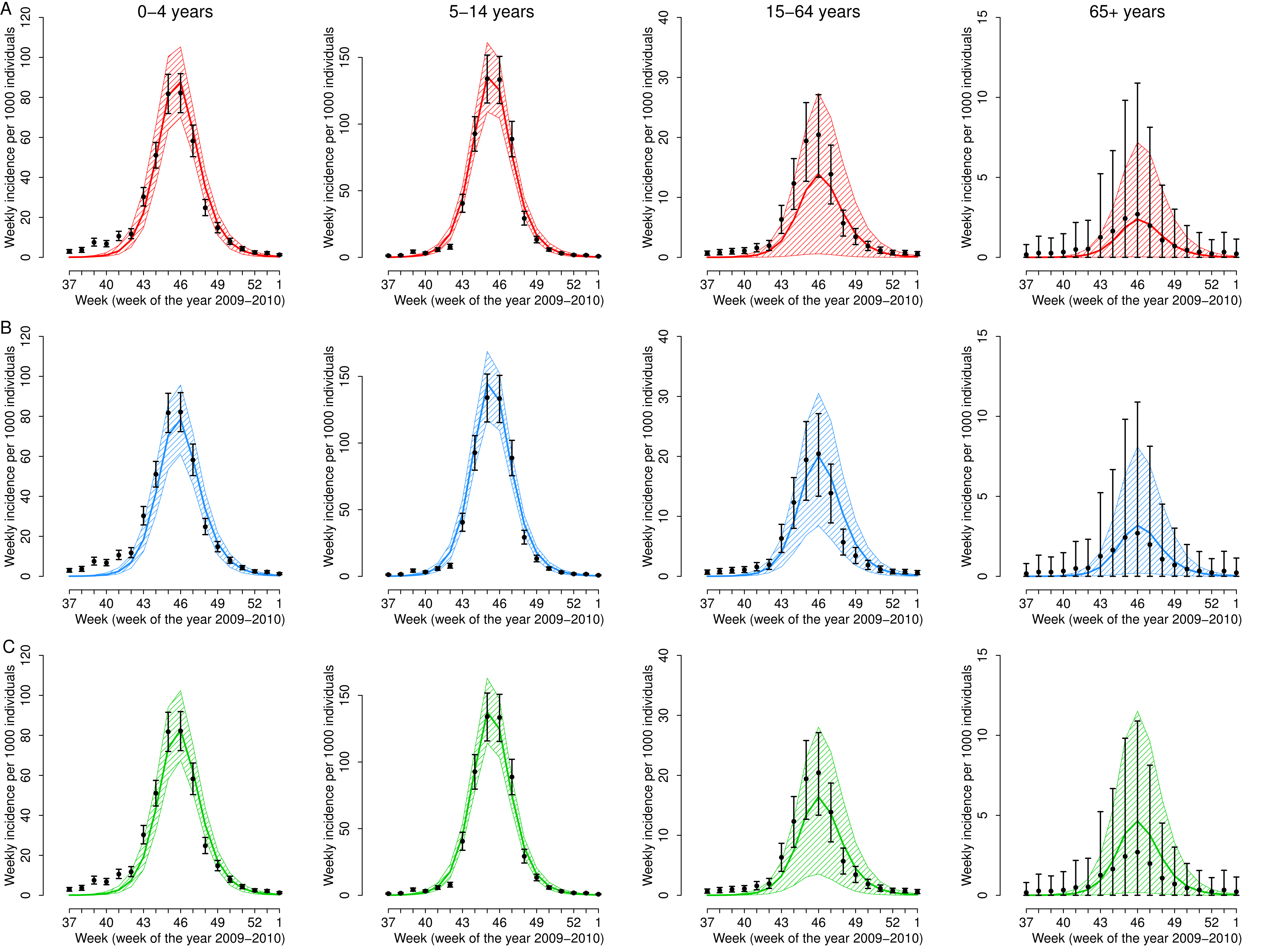

Supplement: Figure S3 — Model fit to rescaled weekly incidence data, assuming to be seropositive when titre is ≥40 and pre-existing full immunity as derived from the analysis of pre-pandemic sera. Average model prediction (colored line) and 95% CI (colored shaded area) and rescaled weekly incidence (black dots) with 95% CI (vertical black lines) in the four age groups. (A) Predictions obtained by assuming contact matrix CM1 [39]. (B) Predictions obtained by assuming contact matrix CM2 [38]. (C) Predictions obtained by assuming contact matrix CM3 [37]. (TIF) [file pone.0074785.s003.tif]

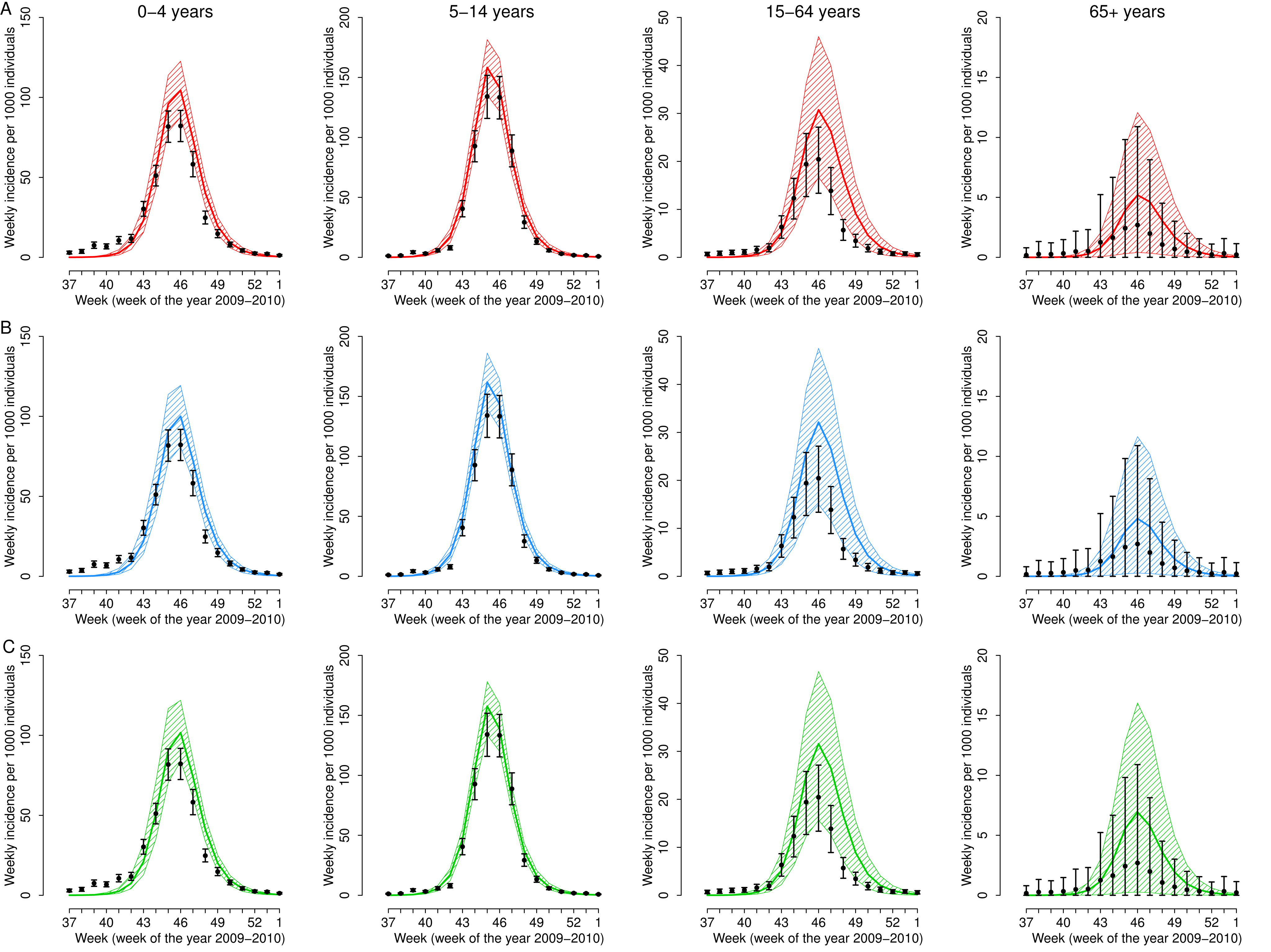

Supplement: Figure S4 — Model fit to rescaled weekly incidence data, assuming to be seropositive when titre is ≥10 and pre-existing full immunity as derived from the analysis of pre-pandemic sera. Average model prediction (colored line) and 95% CI (colored shaded area) and rescaled weekly incidence (black dots) with 95% CI (vertical black lines) in the four age groups. (A) Predictions obtained by assuming contact matrix CM1 [39]. (B) Predictions obtained by assuming contact matrix CM2 [38]. (C) Predictions obtained by assuming contact matrix CM3 [37]. (TIF) [file pone.0074785.s004.tif]
